# Are we Aware of what we are, we are what we Eat- An Epidemiological Survey

**DOI:** 10.5005/jp-journals-10005-1003

**Published:** 2008-12-26

**Authors:** Manpreet Kaur, Amitha M Hegde

**Affiliations:** 1 Postgraduate Student, Department of Pedodontics and Preventive Children Dentistry, AB Shetty Memorial Institute of Dental Sciences, Mangalore, Karnataka, India; 2 Professor and Head, Department of Pedodontics and Preventive Children Dentistry, AB Shetty Memorial Institute of Dental Sciences, Mangalore, Karnataka, India

**Keywords:** Junk food, Television, Nutritional behavior, Children.

## Abstract

The lure of convenience in addition to good taste gets people to junk food addiction. With the advent of television even in the remotest areas people have become more aware of the fast food items available. Children watch television where fast food continues to dominate the food advertisements viewed by children. The easily available fast food in and around school campus and with little knowledge of the far reaching effects of these food items consumed, children fall an easy prey to junk food. Children are unaware of the food they eat and the effects it has on their growth and development. This survey reflects the large percentageof children who surrender to the temptation junk food consumption with little knowledge of its far sighted effects on their health.

## INTRODUCTION

Childhood years are a time of steady growth; good nutrition is a high priority. Metamorphosis of food habits has led to the replacement of nutritious food by things that are tasty, convenient, in vogue-junk food. Food high in salt, sugar, fat or calories and low nutrient content is called junk food. Junk foods provide suboptimal nutrition with excessive fat, sugar, or sodium per kcal.^1^

Such poor diets can slow growth, promote obesity, sow the seeds of diseases like diabetes, hypertension, cardiac problems, osteoporosis, decayed teeth. The role of oral health in overall health reaches well beyond dental caries. Resulting pain due to dental decay leads to seeking of comfort in choice of food that is soft and refined than harder natural food creating a vicious circle.^2^ An increase in the energy density of food consumed, a decrease in satiety, passive over consumption is a significant outcome. 3 Artificial food colors cause learning disabilities due to lapses in concentration.^4^

Convenience, fast foods and sweets continue to dominate food advertisements viewed by children. Advertised foods exceed recommended daily values of fat, saturated fat, and sodium, yet fail to provide fiber and certain vitamins and minerals. 5 Vast majority of working parents with school age children get less time to spend with their children so the traditional food skills are not passed on to them. For children who have less vision of far reaching effects fall prey to junk food.^6^

Changes in our society have intensified the need for food skills to the extent that they must become a part of the child’s basic education for good health and survival. Children are not aware of what they eat affects how they grow, feel and behave, hence the need for the study.

The aim of the study was to determine the prevalence of consumption of fast food among children and their knowledge regarding the food they eat.

## DESIGN

A total population from ten different schools comprising of 2636 children aged between 4 and 15 years from rural schools in and around Mangalore were included in the study.The primary hypothesis was that subjects in the rural areas consumed fast food more due to the advent of television in the remotest areas leading to promotion of such foods through television.

In the study children provided information on their dietary intakes. Knowledge regarding the type of food consumed by them was obtained by a questionnaire in the local language. The study also sought information as to from where each food was obtained and if the advertised food was included in the diet.

**Fig. 1:**
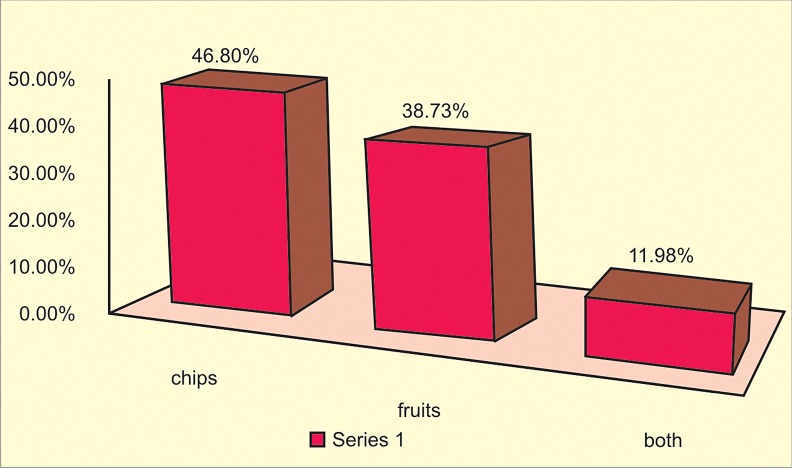

**Fig. 2:**
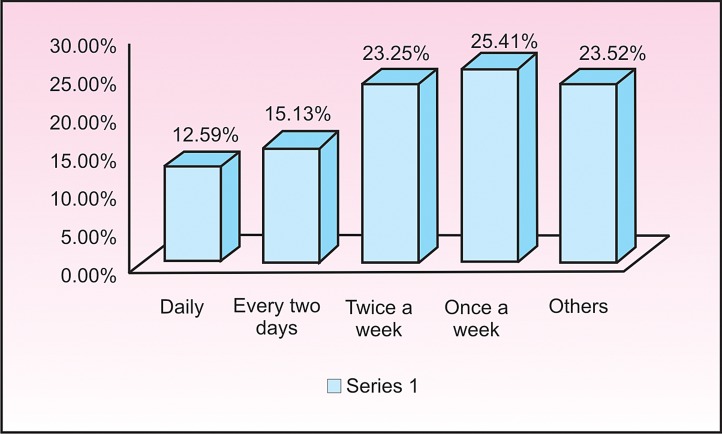

## RESULTS

Of the screened population nearly 65% were males and 35% were females. Fast food prevalence was high among all age groups and both the genders. Children as fast food consumers consumed less of fresh fruits and vegetables with less of fluid milk intake (Fig. 1). Nearly 60% of these children consumed fast food on a daily basis.

All the children had an inclination for snacks and 25% skipped meals for fast food. 58% of the children were advised by their parents not to have these foods.

Inspite of advising the children, 75% of the parents bought snacks for their children at least once a week. 23.52% of the children purchased snacks for themselves as these were readily available in the school premises (Fig. 2).

More than 50% of the children watched fast food being advertised on the television. Candy, sweets, breads, fast foods were advertised more frequently with little representation of fruits and vegetables. As high as 70% were not aware of the nutritional content of this food consumed by them.

## DISCUSSION

Fast food has become a prominent feature of the diet of children in India and, increasingly, throughout the world. More than half of the Indian population is rural and so a proper understanding of their cultural attitudes is a must before any modification of habits is done. Many studies have examined the effects of fast-food consumption on any nutrition or health-related outcome. This epidemiological study comprised screening of two thousand and six hundred children of age 4-12 years depicting a high prevalence of consumption of junk food by children in rural areas. Children of younger age groups were influenced by the answers of the other children.

Some previous studies have pointed out junk food eating gives more total energy and poorer diet quality. 1 Children and junk food have a strange affinity to each other and this addiction is made obvious by the percentage of children fond of it. Nearly 50% of children had a daily consumption of junk food without realising the ill effects of it on their health. Junk food does not provide essential nutrients but satisfies the appetite. Nutritional requirements are not met with, so children may feel weak. The screened children admitted not to have skipped meals for eating junk food. Eating in between meals is one of the causes of unwanted obesity.^3^ With over sustained periods of junk food eating, blood circulation drops due to fat accumulation, obesity a common problem which has taken its toll along with malnutrition.^6^

Statistics of the study show that parents themselves purchased fast food for their children atleast once a week which could be attributed either to the likeness expressed for junk food or the undermining of food habits by the busy jet age setting in. Vast number of children purchased these unhealthy items themselves with very few knowing that these choices were unhealthy. So, adults occupy a centralposition in the process of modification of nutritional behavior.^2^ Student scores regarding the question if their parents advised them not to eat fast food does not relate to the purchase of such food by parents themselves. Parents are to be warned of the dangers of giving their young children drinks, sweets and cakes containing specified artificial additives. Findings confirm their link with hyperactivity and disruptive behavior. Junk food diet is a major cause of heart diseases as pointed out by many studies. High cholesterol from junk food strains liver and damages it eventually.^4^,^7^

In Indian scenario, improved marketing strategies and increased transport facilities have brought food materials like bread and chocolates to even the remotest villages. Television is one such medium of propagating many of these food items.

Awareness on junk food is lacking dramatically in every part of society. Noticed in a large percentage of children was daily consumption of more than one chocolate. There is an obvious predilection for food rich in sugar and energy not meeting with other nutrient requirements.^3^ This was strongly propagated by television advertisements.

Students attending schools of a higher socioeconomic status showed an upsurge in this ratio. Most of the children believed that these advertisements propagated food materials which were healthy.

## CONCLUSION

Children in the rural schools liked junk food but they preferred to have these in between meals. Parents bought fast food items for the children and majority of children bought it for themselves as it was readily available around. Children had such food items almost daily and parents were aware of it.

Majority of the children watched advertisements on television and believed that the food advertised was healthy.

## RECOMMENDATIONS

- First and foremost step to be taken is to create awareness.
- Prohibit fast food advertisements and promotions directed to children on television. Consumers need more guidance in making food choices for themselves and their children.
- Nutritious and healthy food habits must be cultivated in children. Even parents and schools can play a part by imparting knowledge about nutrition.
- Education of school children with audio visual aids on the harmful of effects of this junk food eating is highly recommended. Excellent food choices at schools provided in snack machines, stores and cafeterias would foster their consumption. Traditional, Indian diet is balanced with lots of fibrous components and should not be replaced by high refined sugar foods. Components in traditional diets that may favor oral health need to be identified and propagated.
- Communities, schools, legislative bodies, movies, television, and food companies should partner in promoting healthful food choices.
- Potent organizations like World Health Organization should deal with such universal problems aggressively. Develop awareness for fitness.
- Research and survey on a larger scale needs to be carried out and the results made public effectively.
